# The Influence of Sunflower Seed Hull Content on the Mechanical, Thermal, and Functional Properties of PHBV-Based Biocomposites

**DOI:** 10.3390/ma19020268

**Published:** 2026-01-08

**Authors:** Grzegorz Janowski, Marta Wójcik, Irena Krešić, Wiesław Frącz, Łukasz Bąk, Ivan Gajdoš, Emil Spišák

**Affiliations:** 1 Department of Materials Forming and Processing, Rzeszow University of Technology, Powstańców Warszawy 8, 35-959 Rzeszów, Poland; wf@prz.edu.pl (W.F.); lbak@prz.edu.pl (Ł.B.); 2 Department of Organic Technology, University of Split, Ruđera Boškovića 35, 21 000 Split, Croatia; ibanovac@ktf-split.hr; 3 Department of Technology, Materials and Computer Aided Production, Technical University of Košice, Mäsiarska 74, 04001 Košice, Slovakia; ivan.gajdos@tuke.sk (I.G.); emil.spisak@tuke.sk (E.S.)

**Keywords:** PHBV, sunflower seed hulls (SSH), biocomposites, injection molding, dimensional stability, confinement effect, agricultural waste valorization

## Abstract

This paper presents the potential use of sunflower seed hulls (SSH) as a sustainable filler for poly(3-hydroxybutyrate-co-3-hydroxyvalerate) (PHBV) biocomposites. Ground SSH were incorporated into the PHBV matrix at loadings of 15, 30, and 45 wt% via extrusion and injection molding. The Fourier Transform Infrared Spectroscopy (FTIR) analysis indicated the presence of possible interactions between the filler and the matrix. Mechanical testing revealed a significant increase in stiffness, with the tensile modulus increasing from 2.6 GPa for pure PHBV to approximately 4.5 GPa for the composite containing 45 wt% SSH. However, the tensile strength decreased by approximately 10–40%, while elongation at break dropped to 1.0–1.5%, depending on the SSH dosage, respectively. The thermal analysis indicated that high filler contents suppress crystallization during cooling under laboratory conditions in Differential Scanning Calorimetry (DSC) analysis due to the confinement effect. The key practical advantage is the exceptional improvement in dimensional stability with a processing shrinkage reduction of approximately 80% in the thickness direction. Although water absorption increased with filler loading, biocomposites containing 15–30 wt% SSH exhibited the optimal balance of high stiffness, hardness, and dimensional accuracy. These properties make the developed material a promising option for the production of precise technical molded parts.

## 1. Introduction

Biopolymers are biodegradable or non-biodegradable, environmentally friendly alternatives to polymers produced from both renewable sources, e.g., from sugar, starch, lignin, as well as from petrochemical ones [1]. The significant interest in these materials results from their bio-based nature, biocompatibility, and the ability to biodegrade. Due to their advantages, biopolymers are becoming increasingly important in different sectors of the economy, e.g., in medicine [2], in the construction sector [3], and in the food sector for the production of different types of packaging [4]. The global trends show the increasing production of biopolymers every year. It reached 2.18 million tons in 2023 [5], and the total production growth rate is estimated at approximately 17% [6].

The circular economy concept and the promotion of recycling have led to the application of various waste materials as biopolymer fillers, which represents one of the most rapidly developing research trends. Different by-products or wastes might be treated as low-cost and environmentally friendly biopolymer fillers whose application can decrease the overall cost of the biopolymer while improving its properties [7]. A lot of by-products or wastes are applied as a filler in biopolymer composites, e.g., flax [8], sawdust [9], cotton [10], jute [11], walnut shell [12], kenaf fibers [13], biochar [14], coffee grounds [15], rice husk [16], banana fibers [17], waste eggshells [18], coir [19], sisal [20] and sugarcane bagasse [21]. The selection of the appropriate filler depends on its chemical composition, as well as the material morphology. The material availability, as well as the cost of processing and transportation, are also included.

Natural fibers are widely used in the production of biocomposites. The addition of fibers in proper dosages can improve the mechanical and thermal properties of biocomposites successfully while also decreasing their production cost. In ref. [22], the mechanical properties of polylactic acid (PLA)-sisal and PLA-jute biocomposites were examined. The increase in the maximum strength and the bending properties was noted here. The increase in tensile and flexural strength, as well as the tensile modulus, after the addition of hemp fibers as a filler to the PLA matrix, was shown in ref. [23]. Thermal and selected mechanical properties of biocomposites produced with the use of PHBV and Miscanthus biocarbon were reported in ref. [24]. Selected mechanical properties of PHBV-based biocomposites containing hemp, flax, and wood fibers as fillers were examined in ref. [25].

Recently, the application of different biomass and biomass waste in the production of biocomposites has attracted increasing attention. The sunflower biomass by-products, particularly sunflower seed hulls, play a special role among all kinds of biomass. Sunflower seed hulls (SSH), which are a by-product of sunflower oil extraction, are a waste material that poses a serious utilization challenge. The worldwide sunflower production is estimated at 47.34 million tons annually [26]. It gives SSH annual production at the level of 54 million tons [27]. The seed consists of the core and the hull, which constitutes approximately 45–60% of the total seed weight depending on the sunflower species [28]. The high weight of husk in the total sunflower seed weight causes a huge amount of hulls to be generated annually. For example, the data shows that over 112,000 tons of SSH are produced annually in the United States [29].

However, the low commercial value of SSH resulting from their chemical composition and low bulk density causes a serious problem with their industrial application. Examples of the use of SSH as a natural fertilizer [30] or as a low-fat addition to animal food are described in ref. [31]. Due to the high energy value (17 MJ/kg) and the low water content (below 9%), SSH is also considered a potential fuel source [32]. The works focused on the production of pellets from SSH, and their ashes are also known [33]. In ref. [34], SSH is used for the production of different active materials. Although the data show that less than 50% of the SSH produced are recycled, and the rest of them are stored in landfills [35]. It causes the need to identify new applications for SSH that ensure their effective utilization in line with environmental requirements.

Sunflower seed hulls (SSH) are one of the main sources of natural fibers. They have higher content of fiber, neutral detergent fiber (NDF, 70–85%), acid detergent fiber (ADF, approximately 50%), and calcium (1.47%) in comparison to other fiber sources [36]. It has been noted that SSH consists of 31–51% of cellulose, 13–28% of hemicellulose, 20% of lignin, 4–6% of proteins, and 5% of lipids [36], depending on the harvesting time and climate conditions. The content of cellulose, hemicellulose, lignin, and proteins might significantly influence the hardness, stiffness, and adhesion of the filler to the matrix.

Due to their fiber structure and beneficial composition, SSH might be used as a filler for the production of biocomposites. There are numerous works in which SSH is incorporated as the filler in the polymer matrix. In ref. [37], the biocomposite produced from high-density polyethylene (HDPE), SSH, and barley straw was tested. It was noted that the addition of SSH in the amount of 5–25 wt% had a positive effect on the elastic strength properties of the biocomposite obtained. The application of 20 wt% SSH in the polypropylene (PP) was described in ref. [38]. The incorporation of SSH into the polymer matrix resulted in an increase in flexural strength and tensile modulus compared to pure PP. In other work [39], SSH was added as a filler to the PLA matrix in order to increase the mechanical properties of the biocomposites produced. The influence of SSH addition to different polymer matrices, PLA and polypropylene (PP), in dosages of up to 60 vol.%, was tested in ref. [40]. While a reinforcing effect has been consistently observed in various matrices, research on SSH as a filler remains limited compared to other lignocellulosic materials. In particular, there is a distinct scarcity of studies concerning the incorporation of SSH into a PHBV matrix. This work is among the first to systematically investigate the processing shrinkage and dimensional stability of biocomposites with high filler loadings (up to 45 wt%), which is critical from a materials engineering perspective. Furthermore, this study provides original empirical data on crystallization kinetics, thermal stability, and degradation mechanisms specifically for the PHBV–SSH system, addressing the limitations of existing literature, which is primarily confined to basic mechanical characterization. Consequently, this paper bridges the gap in current knowledge by presenting a comprehensive, multi-faceted evaluation of the thermal, mechanical, and processing properties of injection-molded PHBV–SSH biocomposites.

This paper presents a comprehensive and multifaceted analysis of injection-molded PHBV–SSH biocomposites with the first systematic assessment of their different properties. The aim of this work is the assessment of the influence of SSH content on selected mechanical, thermal, functional, and processing properties of PHBV–SSH biocomposites. The SSH loadings of 15, 30, and 45 wt% were applied here. The hardness, impact tensile strength, shrinkage of molded parts, and water absorption were measured. The crystallization behavior and thermal stability using both Differential Scanning Calorimetry (DSC) and Thermogravimetric Analysis (TGA) were also evaluated here. The identification of functional groups and the assessment of material degradation were carried out using the Fourier Transform Infrared Spectroscopy (FTIR) method. The results obtained confirm the promising potential of the material with regard to sustainable development and highlight the purposefulness of continued research related to its practical application.

## 2. Materials and Methodology

### 2.1. Materials

**Figure 1:**
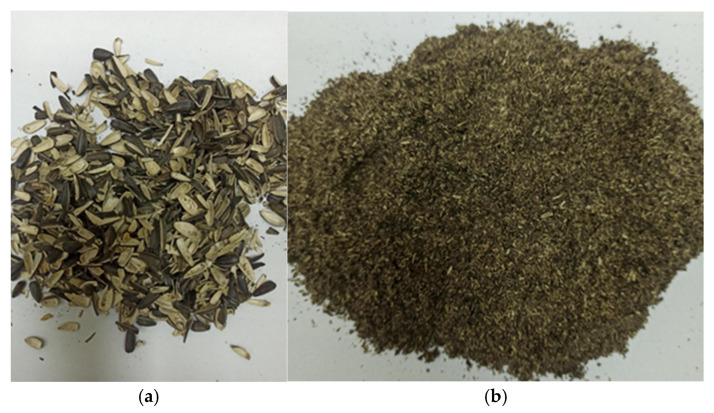
Visual appearance of SSH before (**a**) and after (**b**) milling.

The polymer matrix consisting of PHBV (trade name ENMAT Y1000), sourced from Tianan Biologic Materials Co., Ltd. (Ningbo, China), was used in this study [41]. The material was in the powdered form, which enabled precise dosing and ensured homogenous blending with the SSH filler. Based on data supplied by the manufacturer, the polymer had a density of 1250 kg·m^−3^ and a melt flow index (MFI) from 1 to 5 g/10 min (at 190 °C, 2.16 kg) [42]. The thermal properties included a Vicat softening point of 166 °C [43] and a heat deflection temperature (HDT) in the range of 157–165 °C [44]. These characteristics, typical for PHBV, facilitate processing via standard thermoplastic methods such as extrusion and injection molding. The sunflower seed hulls (SSH) used in the research were obtained from local distributors as a waste product produced during the seed degreasing process (Figure 1a).

The following abbreviations are used in this study for the materials tested:

- SSH—sunflower seed hulls;
- PHBV—poly(3-hydroxybutyrate-co-3-hydroxyvalerate);
- PHBV-SSH15—PHBV-based biocomposite containing 15 wt% of SSH filler;
- PHBV-SSH30—PHBV-based biocomposite containing 30 wt% of SSH filler;
- PHBV-SSH45—PHBV-based biocomposite containing 45 wt% of SSH filler.

### 2.2. Preparation of Raw Mixture

Firstly, SSH was ground using the FW177 high-speed mill (Chemland, Stargard, Poland) with a speed of 2400 rpm. The total grinding time was seventy seconds in order to obtain a homogeneous fraction with fine granulation and a limited amount of fibrous agglomerates. The equal batch portions and periodic cleaning of the grinding chamber were performed to ensure the repeatability of the process and to avoid overheating and the adhesion of lignocellulosic particles. The SSH powder (Figure 1b) obtained after milling was then used as a filler in order to produce three dry mixtures with the PHBV polymeric matrix.

PHBV/SSH weight ratios of 85:15, 70:30, and 55:45 were applied in this work. These filler loadings were selected to evaluate the material behavior across three distinct regimes: the initial reinforcement (15 wt%), the balance between stiffness and processability (30 wt%), and the practical processing limit characterized by saturation effects (45 wt%).

Each of the mixtures was prepared in drier-than-normal conditions by mechanical mixing of components using a rotary low-speed mixer (with a speed of 40 rpm) to achieve homogeneous particle distribution within the PHBV matrix.

The mixtures were then dried at 90 °C for three hours using the DZ-2BC laboratory convection dryer (Chemland, Stargard, Poland). The drying conditions were selected to decrease the total humidity of the material, which can ensure the stability of the extrusion process. This step was necessary to avoid the foaming, pressure instability, plasticizing units, and thermal degradation of the biopolymer. The homogeneous and dehydrated composite granulates were then used in the extrusion and injection molding processes.

### 2.3. Manufacturing Process

#### 2.3.1. The Extrusion Process

The extrusion process of both pure PHBV and PHBV–SSH composites containing 15, 30, and 45 wt% of the filler was performed by applying the ZAMAK EHP-25E laboratory single-screw extruder (ZAMAK Mercator, Skawina, Poland) equipped with a screw diameter of 25 mm and the length-to-diameter ratio (L/D) of 25. The extrusion was carried out using a plasticizing unit with an increasing temperature profile towards the head. It ensured the gradual plasticization of the material, the uniform distribution of the filler, and pressure stability during the process [45,46]. The temperature settings were as follows: 145/155/160/160 °C for pure PHBV, 150/155/160/165 °C for PHBV–SSH15, 155/160/165/170 °C for PHBV–SSH30, and 160/165/170/175 °C for PHBV–SSH45. The rotational speed of the extruder screw was selected to maintain the comparable quality and the geometry of extrudates, which were 100, 30, 40, and 50 rpm, respectively (Table 1).

#### 2.3.2. The Injection Molding Process

The injection molding was performed using a BOY 55E injection molding machine (Dr. Boy GmbH and Co. KG, Neustadt, Germany). The dog-bone-shaped specimens, prepared in line with ISO 527-1:2019 [47], were formed for the uniaxial loading test. The same filling strategy for all samples was maintained in order to ensure comparable thermal history and surface quality. The constant volumetric injection speed of 35 cm^3^/s was applied here. The variations in melt behavior were compensated by the proper selection of melt and mold temperatures, as well as the holding and cooling parameters. The constant flow rate allowed for comparison of the materials without considering the influence of variation in shear conditions (Table 2).

### 2.4. Methods

The microscopic analysis was conducted at a magnification of 30×, which allowed for a precise examination of the external structure and shape irregularities. Simultaneously, a quantitative dimensional analysis was performed using the microscope’s integrated digital software (E-MAX v7.0) of Nikon MM 800 measuring microscope (Nikon, Tokyo, Japan). The length and diameter of 30 randomly selected particles were measured to determine the average particle size distribution and the aspect ratio. This non-destructive observation was essential for evaluating the dimensional uniformity of the biomass particles prior to their use as a filler.

The thermal degradation was performed using the non-isothermal thermogravimetry (TGA) in the temperature range of 50–600 °C at the heating rate of 10 °C/min using a PerkinElmer TGA 8000 thermogravimetric analyzer (PerkinElmer, Waltham, MA, USA). The nitrogen flow rate was 40 cm^3^/min. FTIR measurements were carried out using the PerkinElmer Spectrum Two FTIR spectrometer (PerkinElmer, Waltham, MA, USA). The interactions between polymer and filler were assessed. The attenuated total reflectance (ATR) technique with a diamond crystal was applied here. The FTIR spectra (average of 10 scans) were recorded in the wavenumber range of 4000–450 cm^−1^ at a spectral resolution of 4 cm^−1^.

The differential scanning calorimeter DSC Caliris 300 (Netzsch, Sleb, Germany) was used in order to evaluate the thermal effects of adding SSH to PHBV polymer. The samples were placed in a standard aluminum pan and covered with an aluminum lid. All samples were first cooled from 25 °C to −30 °C at cooling rate of 10 °C/min, then held at −30 °C for 5 min, then heated from −30 °C to 230 °C at a scan rate of 10 °C/min, held at 230 °C for 5 min, then cooled from 230 °C to −30 °C at a cooling rate of 10 °C/min, and held at −30 °C for 5 min. Finally, the samples were heated once again from −30 °C to 230 °C at a scan rate of 10 °C/min. All DSC measurements were performed using nitrogen gas with a flow rate of 40 mL/min. The degree of crystallinity (*X*_c_ (%)) was calculated according to the following equation (Equation (1)) [48]:

(1) $$X_{c}(\%)=\frac{{(∆H}_{m}-∆H_{cc})}{\left(\frac{100-wt\%}{100}\right)}\times \frac{100}{∆H_{0}}$$

where *X*_c_ is the crystalline fraction of the matrix, ∆*H*_m_ is the melting enthalpy (J/g), ∆*H*_cc_ is the cold crystallization enthalpy (J/g), wt% is the SSH content by weight, and ∆*H*_0_ is the theoretical fusion enthalpy of 100% crystalline PHBV. In this calculation, 146.6 J/g was used as the value of ∆*H*_0_ [48].

The mechanical performance of the produced biocomposites was assessed using the Zwick Z030 universal testing machine (Zwick/Roell, Ulm, Germany). The uniaxial tensile tests were conducted on dog-bone-shaped samples in accordance with ISO 527-1:2019 standard [47]. The stress–strain curves obtained were then used for the determination of critical mechanical properties, including the tensile strength, Young’s modulus, and the elongation at break.

Material resistance to fracture under dynamic loading conditions was investigated by the tensile impact testing in accordance with the EN ISO 8256:2004 [49] standard. The CEAST 9050 pendulum impact tester (Instron, Norwood, MA, USA) was used in order to precisely quantify the energy absorbed by the sample during rapid failure. The hardness properties were determined using the ball indentation technique in line with EN ISO 2039-1:2003 [50] standard. These measurements were performed in two distinct regions of the tensile specimens: the narrow gauge section (designated as area A) and the wider grip section (designated as area B). The Zwick 3106 hardness testing device (Zwick/Roell, Ulm, Germany) was used for this analysis, which enabled the evaluation of potential local variations in mechanical properties across different zones of the specimen.

The water absorption of samples was examined following the EN ISO 62 [51] standard. Additionally, the linear shrinkage of the dog-bone-shaped specimens was measured in line with EN ISO 294-4:2001 [52] standard, which allowed for the assessment of dimensional stability and changes induced by the processing and cooling stages.

To ensure the statistical reliability of the experimental findings, all mechanical tests and shrinkage evaluations were performed on seven specimens for each material formulation. Based on the collected data, mean values and standard deviations were calculated and summarized in graphical and tabular form in this study.

## 3. Results and Discussion

### 3.1. Microstructural Analysis of SSH Filler

**Figure 2:**
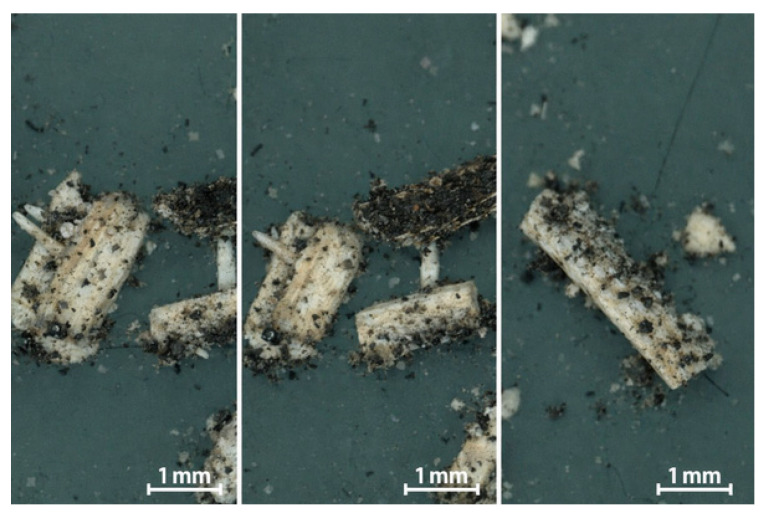
Microscopic images of ground SSH (magnification: 30×). The lighter fibrous particles correspond to the cellulose-rich inner tissue, while the darker lamellar fragments represent the lignified outer layer containing phytomelanin. The visible surface irregularities indicate macroscopic roughness and pores.

Ground SSH containing lignin and cellulose is characterized by a complex structure and morphological diversity. Microscopic observations show that particles have an irregular shape, containing both short fibrous elements and lamellar ones derived from the outer layer of the hulls (Figure 2). Some of the particles also indicate a rough, irregular surface with visible pores and fissures, which are the residue obtained after the separated cell walls. The inner part of the hull, which contains mainly cellulose and hemicellulose, is characterized by a brighter color. The outer part has a darker color and includes cuticular layers that are rich in wax compounds. This structural heterogeneity causes individual particle fragments to differ significantly in both topography and surface wettability, which is important for subsequent interfacial interactions with the polymer matrix.

The particles are characterized by lengths in the range of 0.65–1.53 mm (0.99 ± 0.20 mm on average) and diameters of 0.19–0.52 mm (0.34 ± 0.09 mm on average). It gives a length-to-diameter ratio (L/D ratio) in the range of 2.6–6.4, with the average value of approximately 3.0. These geometrical proportions are characteristic of short lignocellulose fibers, which are used as a filler in composite parts produced by injection molding [53,54]. The fraction with a low value of L/D ratio promotes the uniform dispersion of particles in the matrix and reduces the risk of their excessive damage during processing. It also contributes to the stiffening of composites without the anisotropy of mechanical properties.

Microscopic analysis reveals that SSH particles exhibit a highly developed surface texture characterized by micropores and structural fissures derived from the plant tissue. This morphology differs from the smooth, platelet-like structure often observed in common mineral fillers such as talc. The micropores and structural fissures might promote the mechanical anchoring of SSH particles in the polymer matrix. The presence of numerous hydroxyl groups creates potential sites for hydrogen bonding with the carbonyl groups in PHBV. These interactions might improve interfacial adhesion, particularly in places where the particles are partially plasticized and are pressed into the matrix during the forming process. The outer, darker layer of the hull, which contains lignin and waxy substances, might limit wettability in some places and might result in the formation of weaker interfacial zones [53]. Although the FTIR evidence is consistent with hydrogen bonding, more advanced techniques (e.g., solid-state nuclear magnetic resonance, NMR) might be required to quantify the strength and the density of these interactions.

It is worth highlighting that SSH is a raw material that is characterized by a low bulk density of approximately 0.15–0.25 g/cm^3^ [55] and high porosity, which impedes its dosage and uniform feeding into the extruder. The fraction with a relatively uniform size distribution was obtained after grinding and sieving. However, the presence of fine dusty particles and flaky cuticle parts was noted. This fraction, which might be unfavorable from the rheological point of view, can act as local crystallization initiators. This phenomenon has also been observed in other biopolymer-based lignocellulosic composites [53].

The mechanical testing results, specifically the significant increase in tensile modulus and hardness accompanied by a reduction in elongation at break (discussed in Section 3.7 and Section 3.9), confirm that SSH acts as a rigid particle filler within the PHBV matrix. The addition of SSH particles to the PHBV matrix increases stiffness and dimensional stability, with a simultaneous slight decrease in impact strength and elongation at break. It is caused by the phase discontinuity and by the limited deformability of the filler, which is confirmed by the previous research on epoxy and elastomeric materials containing sunflower husks [56,57].

The literature review shows that SSH are cheap and stable reinforcing component. Due to the presence of lignin and hemicellulose, the lignocellulosic structure influences the crystallization of biopolymers in a positive way, e.g., for PHBV, PLA, and PBS [53]. On the other hand, the heterogeneous surface structure, differences in polarity, and the presence of waxy substances might require additional surface treatments, such as mechanical, oxidative, or chemical activation, for improving adhesion and dispersion homogeneity in the polymer matrix. Sunflower seed hulls (SSH) are a lignocellulosic filler with a complex microstructure that contains zones rich in cellulose, lignin, and waxes. The fraction obtained after the milling is characterized by an irregular shape and significant roughness. The properties can facilitate mechanical coupling with the polymer matrix, but also lead to local interface inhomogeneity. This morphology is advantageous for the extrusion and injection molding processes, ensuring uniform particle dispersion within the matrix and good dimensional stability of the molded parts.

### 3.2. Biocomposite Extrusion Analysis

The decrease in rotational speed with increasing content of the filler was necessary to compensate for the growth of viscosity and torque (Table 1). It enabled the avoidance of excessive shear heating, as well as ensured a stable and continuous flow. For example, the application of a rotational speed of 30 rpm allowed for smooth processing and even flow without pressure pulsations for the PHBV–SSH15 composite. Such a rotational speed also helps preserve the particle morphology. The PHBV–SSH45 composite, which has the highest viscosity and the greatest flow resistance, required an increase in the screw rotational speed to 50 rpm. It allows for sufficient and full plasticization and appropriate process performance while maintaining a uniform flow [45,46,58]. On the other hand, the increase in rotational speed to up to 50 rpm might result in higher particle damage. The increased temperature gradient toward the head ensured the gradual plasticization of the PHBV matrix and improved the dispersion of SSH particles within the matrix [46]. The lower rotational speeds for composites with a smaller filler content facilitated the preservation of particle morphology. On the other hand, the higher rotational speed enhanced the wetting and dispersion of the filler in composites containing 45% SSH. The reduction in the formation of voids and surface defects was also noted. The foaming, pulsation, and pressure surge phenomena were not observed. It confirms the effective drying of the components and the proper selection of processing parameters [59]. The proper drying of both PHBV and the filler is essential to maintain the stability of the extrusion process and to prevent hydrothermal degradation of the matrix. It leads to a decrease in the molecular weight and a deterioration of the mechanical properties of products obtained [60].

From a rheological point of view, the increasing filler content results in an increase in apparent viscosity and in a reduction in the mobility of the PHBV chain segments, which promotes flow behavior characteristic of pseudoplastic fluids [58]. The presence of lignocellulosic particles also increases the internal friction [58,61]. However, the appropriately selected temperatures and rotational speeds provide sufficient shear stress to achieve a homogeneous mixture without the risk of degradation or thermal discoloration of the matrix.

The application of the optimal process parameters enables the obtaining of extrudates with a homogeneous structure, a stable diameter, and a smooth surface, regardless of the filler content [59]. This ensured that the mechanical, thermal, and morphological properties of the composites could be compared without the influence of variations in processing quality. The established range of extrusion parameters (145–175 °C, 30–50 rpm) was suitable for the stable processing of PHBV–SSH biocomposites, ensuring reproducibility, preservation of filler morphology, and the absence of degradation phenomena.

The extrusion of PHBV biocomposites containing SSH was characterized by high operational stability in a wide range of filler content (15–45 wt%). The temperature gradient and proper regulation of the rotational screw speed enable smooth plasticization of the material and effective particle dispersion. It also ensures the maintenance of continuous flow, which is essential to preserve the properties of the final product. The authors’ observations are consistent with results obtained by other researchers who reported an increase in mixture viscosity with increasing filler content [45,46,58,61]. It requires the precise adjustment of process parameters, particularly temperature and screw rotational speed. Gallos et al. [46] noted that the extrusion of composites containing natural fibers is characterized by increased flow resistance and susceptibility to different instabilities, particularly at high filler concentrations. They recommend the proper selection of temperature profile and the reduction in screw rotational speed to prevent matrix degradation [46].

The increase in SSH content in biocomposites to 45 wt% also required raising the temperature to 175 °C and the screw rotational speed to 50 rpm. It enables proper plasticization and also promotes better wetting and dispersion of the particles within the matrix. A similar phenomenon was described by Srubar et al. [62]. It was observed that the improved wetting and dispersion in PHBV–wood flour composites with high filler content (28–46%) enhance material homogeneity and increase composite stiffness. On the other hand, they might result in a reduction in tensile strength due to matrix embrittlement [62]. Masanabo et al. [63] showed that the extrusion process influences the crystalline morphology of PHBV. The lignocellulosic additives lead to the formation of a heterogeneous crystalline structure, which affects the mechanical and thermal properties of the final product. Proper selection of temperature and rotational speed also enabled the control of crystallization, preventing thermal degradation and ensuring the favorable mechanical properties in this work.

Flow stability and lack of pulsation observed in the work indicate good homogenization of the PHBV–SSH mixture. Hoffmann et al. [64] noted that the addition of lignocellulosic fillers increases the risk of forming structural discontinuities. However, it might be controlled by optimizing thermal conditions and shearing in the extruder zones. Additionally, proper dispersion of the filler can contribute to reducing surface defects.

The transition of the PHBV–SSH mixture towards the pseudoplastic behavior with increasing content of filler noted in this work is also characteristic of biocomposites containing plant fibers. Chan et al. [58] emphasized that it is not possible to avoid the increase in internal friction and elastic component in such mixtures. Nevertheless, it might be advantageous in terms of rheological control and formation of stable extrudates.

### 3.3. Injection Molding Analysis

In this work, the melt and mold temperatures for pure PHBV were 167 °C and 60 °C, respectively (Table 2). The packing and cooling time was 25 s, and the packing pressure was 30 MPa. It was a reference point characterized by low viscosity and moderate crystallization, which enabled the rapid solidification and stable geometry of the specimen. The increase in apparent viscosity and the rapid nucleation of PHBV crystallites were noted with increasing SSH content in biocomposites. The melt temperature was increased by three to five degrees, depending on the filler content: 170 °C for PHBV–SSH15, 175 °C for PHBV–SSH30, and 180 °C for PHBV–SSH45, respectively. It was performed to maintain the same injection speed without increasing flow resistance and to prevent premature freezing in thin sections. Simultaneously, the mold temperature increased to 65, 70 and 75 °C, respectively. The higher mold temperature compensated for the accelerated filler-induced crystallization and stabilized the flow front. It also reduced the risk of short shots while improving surface replication.

The parameters of the packing phase were selected based on the differences in compressibility and volume shrinkage. The packing pressure of 45 MPa was applied to PHBV–SSH15, PHBV–SSH30 and PHBV–SSH45. The packing time was extended to 30 s. The higher packing pressure allowed for compensation of material loss during crystallization and cooling. The packing time was maintained at least until the gate solidified, which reduced the risk of the formation of voids. The extension of the cooling time to 30 s was necessary due to the higher mold temperature, and the higher content of solid fraction after crystallization led to the slower heat dissipation. This combination of packing and cooling parameters ensured stable geometry, consistent part weight, and a smooth surface in the whole range of filler content.

From an engineering point of view, maintaining constant injection speed and the gradually increasing both melt and mold temperatures with increasing SSH content is consistent with the rheology and kinetics of PHBV crystallization in the presence of lignocellulose particles [65]. SSH particles act as nucleation centers and accelerate the crystallization process [66], which shortens the time to freezing of the flow. The increased mold temperature extends the time available for pressing and the pressure equalization in the cavity, ensuring that the composite parts do not suffer from underfilling or excessive shrinkage gradients compared to the pure PHBV. At the same time, a moderate increase in the melt temperature decreases the viscosity during filling, which facilitates the particle wetting and distribution without introducing excessive shear that could result in degradation of the matrix [67].

The set of parameters applied in this work gives a clear process window. For pure PHBV, a temperature of 167 °C in the nozzle and 60 °C in the mold cavity ensures a fast cycle with the minimal clamping force. The increase in temperature by approximately 5 °C and the growth of pressure to 45 MPa, with a packing and cooling time of 30 s, stabilized the filling process and compensated for the shrinkage of PHBV–SH15. Maintaining temperatures of 175 °C and 70 °C enables comparable geometry of samples at the same injection speed. Temperatures of 180 °C and 75 °C, together with the same packing force, packing time, and cooling time, counteracted premature crystallization and allowed for the production of goods with the quality comparable to the other batches. In all cases, the parameters were selected in such a way that material differences do not affect the processing artifacts, allowing for a reliable comparison of the mechanical properties of samples.

The described injection molding process and the adjustment of parameters to the increasing filler content are consistent with results reported in the literature concerning biocomposites produced with the use of PHBV and other biodegradable polymers. Masanabo et al. [63] indicated that the content of cowpea fibers in PBSA/PHBV composites accelerated the crystallization process significantly. It also required increasing both the melt and mold temperatures to maintain stable flow and to prevent premature solidification of the material in the cavity. It was achieved by the gradual adjustment of the temperature profile and clamping pressure, similarly to the PHBV–SSH composites.

Amaro et al. [68] also described the influence of lignocellulosic additives on the injection molding of composites produced based on the PHBV matrix. They proved that the increasing filler content in biocomposites leads to an increase in flow resistance and complicates the replication of parts. However, it might be compensated successfully by the application of the higher mold temperature and the extended packing time. The approach was also used in this work. In other work, Montanes et al. [69] described biocomposites containing thyme waste as a natural filler. They reported a significant influence of solid fraction content on the change in rheology and the need to optimize clamping and cooling phases. The results reported in ref. [69] show that longer clamping time and higher pressure are necessary to avoid underfilling in samples and to improve the surface quality in biocomposites with high filler content. It is consistent with the approach used for PHBV–SSH30 and PHBV–SSH45 specimens. The research carried out by Mengeloğlu and Çavuş [70] concerning the injection molding of TPU composites with lignocellulosic fillers confirms that a sufficient increase in melt and mold temperatures and an extension of cooling time are necessary for biocomposites with higher fiber content. Otherwise, shrinkage and structural defects might occur. Similar issues have been effectively eliminated in this work by controlling clamping and cooling times.

The literature analysis indicates that the strategies presented in this work are consistent with the current state of knowledge. The set of processing parameters used in the work enables the effective processing of PHBV biocomposites containing SSH as a filler while ensuring good quality of specimens and comparability of results between particular batches.

### 3.4. Fourier Transform Infrared (FTIR) Analysis

**Figure 3:**
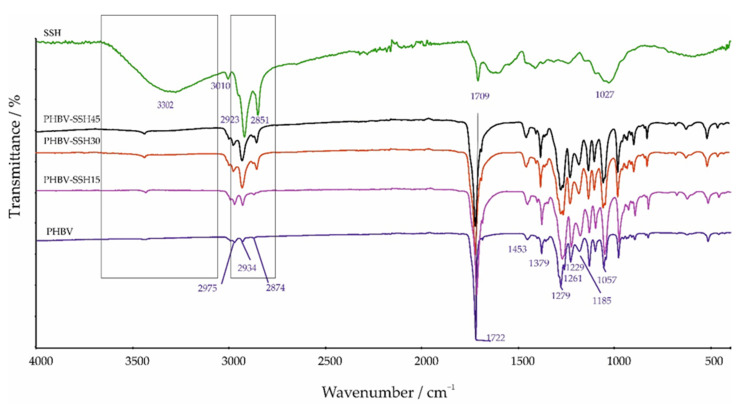
FTIR spectrum of all analyzed samples.

Knowledge of the relationship between composition, morphology, and properties of composites is crucial for designing materials with tailored characteristics. The analysis of FTIR spectra provides valuable insights into potential interactions between the polymer matrix and the filler. Figure 3 shows the FTIR spectra of pure PHBV, SSH, and PHBV–SSH composites.

The FTIR spectrum of pure PHBV exhibits several absorption peaks in the range of 2975–2874 cm^−1^, corresponding to C–H stretching vibrations. Specifically, the peak at 2975 cm^−1^ is attributed to CH_3_ asymmetric stretching modes. The peak at 2934 cm^−1^ corresponds to CH_2_ asymmetric stretching modes and the peak at 2874 cm^−1^ represents CH_3_ symmetric stretching modes [71]. The peak at 1722 cm^−1^ is assigned to the C=O stretching vibration of the ester group and is characteristic of the molecular chain with a highly crystalline structure [72]. Additional peaks are observed at 1380 and 1453 cm^−1,^ which correspond to C–H bending vibrations. Absorption bands at 1261 and 1279 cm^−1^ are associated with C–O stretching vibrations in the ester, while characteristic peaks for C–O–C stretching vibrations appear in the range of 1229–1060 cm^−1^ [73,74].

Sunflower seed hulls (SSH) are lignocellulosic materials primarily composed of cellulose, lignin, and hemicellulose, each contributing characteristic functional groups. The FTIR spectra of SSH display broad absorption bands in the range of 3600–3100 cm^−1^, which correspond to hydroxyl groups. Peaks at 2923 and 2852 cm^−1^ are attributed to the C–H bond stretching vibrations of SSH. A minor peak at 3010 cm^−1^ is associated with the C=C–H stretching vibration of fatty acids. The peak observed at 1709 cm^−1^ corresponds to the symmetric C=O stretching of carbonyl groups, while the peak at 1027 cm^−1^ is indicative of C–OH stretching vibrations associated with alcohols and carboxylic acids [75]. The comparison of the FTIR spectra of PHBV/SSH composites with those of pure SSH and PHBV shows that the hydroxyl stretching vibrations of SSH at 3300 cm^−1^ become less visible and broader, suggesting the formation of hydrogen bonds between the hydroxyl groups of SSH and the C=O groups of PHBV. Also, the small peak at 3010 cm^−1^ originating from fatty acids is shifted to a lower wavenumber of 3007 cm^−1^ after incorporation into PHBV, indicating the involvement of carboxylic acids in bonding with the polymer matrix. In the FTIR spectra of PHBV/SSH composites, an overlap of the spectral bands characteristic of pure PHBV and SSH can be observed in the C–H stretching region. However, the addition of filler continuously shifts the C–H stretching band of PHBV from 2934 cm^−1^ to 2932 cm^−1^ for the sample with 15 wt% and to 2928 cm^−1^ for the sample with 45 wt% filler, indicating the interaction between the filler and the polymer matrix. Furthermore, the increase in filler content leads to an increase in intensity, as well as a broadening and changes in the shape of these absorption bands.

It was also noted that the C=O stretching vibration bands at 1722 cm^−1^ of PHBV broaden and shift towards lower wavenumbers with the increasing filler content (Figure 3). This observation, together with the earlier observation of the broadening of the hydroxyl stretching vibrations of SSH at 3300 cm^−1^ in the presence of PHBV, might suggest the formation of hydrogen bonds between the hydroxyl groups of SSH and the –CO– groups of PHBV (see Figure 3). However, it must be emphasized that FTIR analysis provides only qualitative, indirect evidence of interactions between PHBV and SSH. Consequently, a complete quantification of the interfacial adhesion strength would require complementary research, such as micromechanical testing, dynamic mechanical thermal analysis (DMTA), or surface energy measurements, which were beyond the scope of this study.

Another important band shift from 1279 in pure PHBV to 1272 cm^−1^ in the composite containing 45 wt% SSH corresponds to C–O stretching. Further shifting is seen in bands associated with C–O–C stretching vibrations from 1229 cm^−1^, 1185 cm^−1,^ and 1057 cm^−1^ in pure PHBV to 1225 cm^−1^, 1179 cm^−1^, and 1052 cm^−1^ in the composites with 45 wt% SSH, respectively. These spectral shifts provide further qualitative evidence consistent with intermolecular interactions between the PHBV matrix and the SSH filler.

### 3.5. Non-Isothermal Thermogravimetry (TGA)

**Figure 4:**
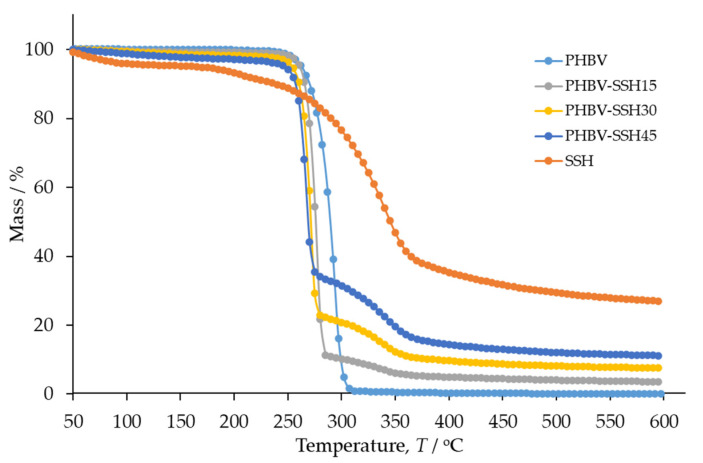
Thermogravimetric (TG) curves for all analyzed samples.

**Figure 5:**
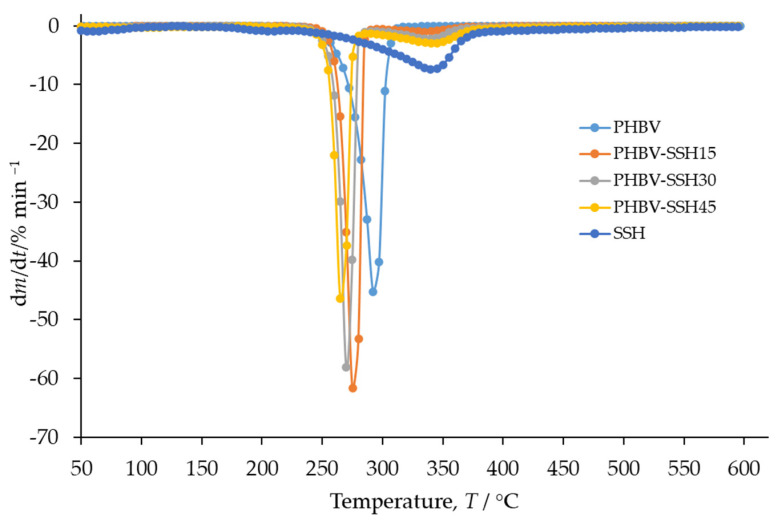
Derivative thermogravimetric (DTG) curves for all tested samples.

Thermogravimetric Analysis was performed to evaluate the influence of the SSH content on the thermal stability of PHBV. Thermogravimetric (TG) and derivative thermogravimetric (DTG) curves of PHBV, SSH, and PHBV–SSH composites are presented in Figure 4 and Figure 5. The corresponding characteristics are summarized in Table 3 and Table 4. The TG and DTG curves of PHBV exhibit a single degradation step, observed as a peak on the DTG curve within the temperature range of 240–320 °C. The onset degradation temperature (*T*°) of PHBV is 279 °C (Table 3), which is consistent with previous studies [76]. According to the literature, the thermal degradation of PHBV follows a random chain scission mechanism involving *β*-hydrogen elimination (a six-membered ring ester degradation process), leading to the formation of olefins and oligomers [76].

The TG curve of SSH (Figure 4) shows an initial mass loss at *T*° = 41 °C, associated with adsorbed water (4.9% of the sample, according to TGA results) and two main degradation steps with *T*° values of 181 and 297 °C (Table 4). According to the literature, the pyrolysis of hemicellulose and cellulose occurs rapidly, with hemicellulose undergoing significant mass loss between 220 and 315 °C and cellulose between 315 and 400 °C. In contrast, lignin is more resistant to degradation, showing the weight loss over a wide temperature range (160–900 °C). As shown in Figure 4, the degradation profiles of the major SSH components overlap, beginning with the slow degradation of lignin and continuing with the more pronounced degradation of hemicellulose and cellulose.

The degradation of PHBV–SSH composites occurs in two stages within the temperature ranges of 220–290 °C and 290–400 °C, respectively. The first mass loss corresponds to an overlap of PBHV degradation and the beginning of SSH one, with onset temperatures shifting to lower values than those of neat PHBV. As the SSH content increases, the onset degradation temperature (*T*°_1_) decreases progressively, with reductions of 11 °C for the composite containing 15 wt% SSH and 18 °C for the composite containing 45 wt% SSH (Table 3). A similar trend is observed for the temperature at the maximum degradation rate (*T*_max1_), which decreases by 16 °C for the composite with 15 wt% SSH and by 27 °C for the composite with 45 wt% SSH.

The TGA of composites shows that the second degradation stage corresponds to the second degradation step of the filler. The degradation of SSH in this temperature range shows increased temperatures *T*°_2_ in comparison with those for neat SSH. The maximum degradation rate of PHBV (*R*_max1_) increases with the addition of SSH. Only for the sample containing 45 wt% SSH, a reduction in the *R*_max1_ is observed in comparison to other composites. It is probably caused by changes in the degradation mechanism at higher filler contents. Considering the second degradation event, all materials showed similar behavior by increasing *R*_max2_ with higher filler loading. However, the comparison of *R*_max2_ of composites with *R*_max2_ of neat SSH showed a decreased maximum degradation rate for the composites. Table 3 shows that the amount of residual mass, *m*_f_, increases with the addition of SSH filler as lignin leaves a substantial solid residue within this temperature range. In conclusion, the addition of SSH decreases the thermal stability of PHBV. Despite the reduction in decomposition onset temperature by several degrees, the range of processing temperatures for the PHBV–SSH biocomposites was not exceeded. In our injection molding tests, the highest barrel temperature was 180 °C. The degradation temperature for the PHBV–SSH biocomposite with the highest tested dosage of SSH (45 wt%) was approximately 264 °C. Therefore, there is a safety margin of over 80 °C, meaning that thermal degradation does not occur during normal processing. However, degradation may begin with prolonged exposure to high temperatures. From a practical point of view, it is necessary to avoid heating the composite with high SSH content in the machine cylinder for too long to prevent degradation of the organic components of the filler (hemicellulose is less thermally stable and might decompose at temperatures of approximately 220–315 °C). Although the addition of SSH reduces the thermal stability of the material and products made of this composite might have a slightly lower maximum operating temperature than pure PHBV, this is not a significant limitation for typical applications of biopolymers and does not diminish the suitability of the material.

### 3.6. Differential Scanning Calorimetry (DSC)

**Figure 6:**
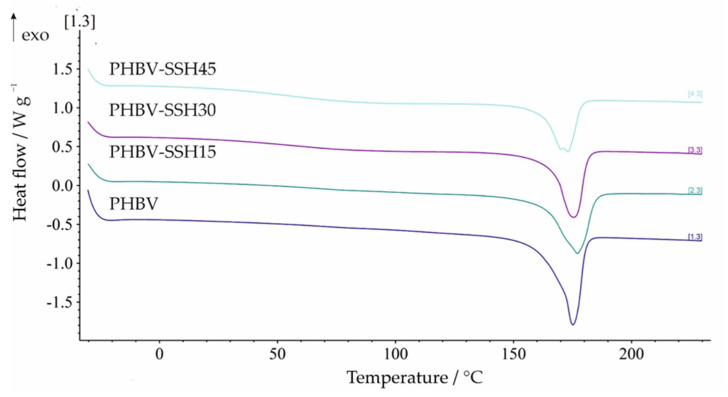
DSC curve of PHBV and PHBV–SSH biocomposites obtained from the first heating scan.

Differential Scanning Calorimetry was performed to examine the effect of SSH on the thermal transitions of the PHBV polymer. DSC heating scans were used to determine the glass transition temperature (*T*_g_), cold crystallization temperatures (*T*_cc_), and enthalpies of cold crystallization (Δ*H*_cc_), as well as melting temperatures (*T*_m_), melting enthalpies (Δ*H*_m_), and to calculate the degree of crystallinity (*X*_c_). Characteristic melting temperatures expressed as the extrapolated onset (*T*_eim_), extrapolated end (*T*_efm_), and peak temperatures (*T*_pm_), as well as melting enthalpies (Δ*H*_m_), were determined from the first and second heating scans. The results obtained from the first heating scan of all samples were analyzed in order to evaluate the influence of the manufacturing process on the properties of molded samples. The DSC curves of the first heating scan of PHBV and all biocomposites with varying SSH content are presented in Figure 6, while the corresponding characteristic melting temperatures, melting enthalpies, and *X*_c_ values are given in Table 5.

In the first heating scan, neither a glass transition region nor cold crystallization peaks were observed (Figure 6). This indicates that the materials attained their maximum achievable crystallinity during the injection molding process, suggesting that the cooling conditions during manufacturing allowed for the completion of the crystallization process prior to the DSC analysis. However, endothermic peaks were observed for pure PHBV, as well as for all biocomposites, corresponding to the melting of the crystalline phase of PHBV. The position and shape of the endothermic peaks vary depending on the filler content. These shifts suggest potential variations in the lamellar thickness and crystal perfection, as the melting temperature of semi-crystalline polymers is inherently linked to the quality and size of the crystalline domains. The DSC curve of neat PHBV exhibits a well-defined endothermic peak with melting temperatures expressed as *T*_eim_, *T*_pm_, and *T*_efm_ appearing at 169.3, 175.2, and 180.7 °C, respectively. The addition of 15 wt% SSH resulted in increased melting temperatures (with the exception of *T*_eim_) compared to neat PHBV, accompanied by a corresponding increase in crystallinity (Table 5). The other two biocomposite materials showed a decrease in melting temperatures relative to PHBV–SSH15, with crystallinity affected by the varying SSH content. Moreover, the bimodal endothermic melting behavior was observed in the sample containing 45 wt% SSH. The lower melting peaks of the bimodal endotherms can be attributed to the melting of thin, less stable crystals formed due to the heterogeneous distribution of the crystals and non-uniform crystal thickness in PHBV caused by the hindrance of SSH, while at the higher temperatures, there is the true melting peak of PHBV [77]. It can be concluded that the melting temperatures and crystallinity of the biocomposites were influenced by various factors such as composite composition and parameters of the manufacturing process.

**Figure 7:**
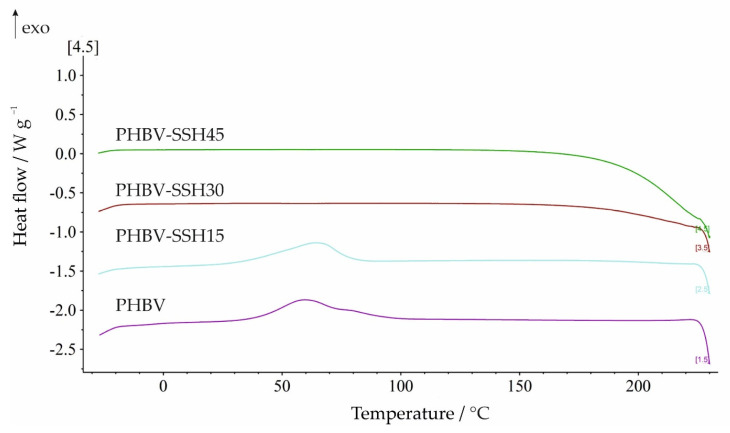
DSC curve of PHBV and PHBV–SSH biocomposites obtained from the cooling scan.

**Figure 8:**
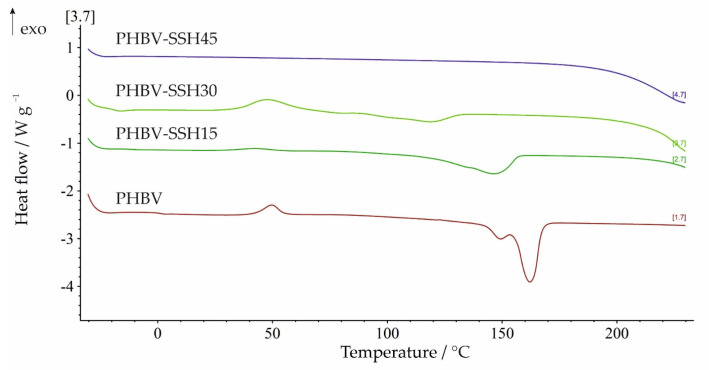
DSC curve of PHBV and PHBV–SSH biocomposites obtained from the second heating scan.

Significant changes caused by the addition of SSH filler are observed from the DSC cooling scan and the second heating scan after erasing the previous thermal history of the material. Results prove once again that the presence of SSH influences the crystallization behavior of PHBV (Figure 7). The characteristic temperatures and enthalpies derived from the cooling scan (the extrapolated onset (*T*_eic_) and endset (*T*_efc_) crystallization temperatures, the peak crystallization temperature (*T*_pc_), as well as enthalpies of crystallization (Δ*H*_c_) and parameters of the second heating scan of the biocomposites were determined and summarized in Table 6. Thus, when comparing the cooling-scan data reported in Table 6, a clear difference between neat PHBV and PHBV–SSH composites emerges. For neat PHBV, crystallization from the melt occurs only partially during the cooling scan and continues during the second heating scan (Figure 8), where crystallization peaks after a clear glass transition step are found. This indicates that the sample remains largely amorphous after the nonisothermal crystallization occurring during cooling and is therefore able to recrystallize from the amorphous phase upon reheating. Temperatures of cold crystallization (*T*_cc_), the extrapolated onset (*T*_eicc_), endset (*T*_efcc_), and crystallization temperatures, the peak crystallization temperature (*T*_pcc_), as well as enthalpies of cold crystallization (Δ*H*_cc_) are listed in Table 6. A similar situation is obtained for the sample with 15 wt% SSH, only the Δ*H*_c_ value during the cooling scan decreases, and later exhibits a decreased Δ*H*_cc_ during the second heating scan. In contrast, at higher SSH contents (30 and 45 wt%), crystallization from the melt is not observed. Only the PHBV–SSH30 sample showed cold crystallization from the amorphous phase during reheating, while the PHBV–SSH45 sample showed a complete absence of crystallization from the melt during cooling and recrystallization during the second heating cycle in DSC analysis. It is crucial to distinguish between the crystallization behavior observed during the rapid non-isothermal DSC cooling (10 °C/min) and the actual microstructure of the injection-molded specimens. While the DSC cooling results indicate a complete suppression of crystallization for high SSH contents due to the confinement effect under these specific laboratory conditions, the injection molding process employed mold temperatures adjusted to the filler content (increasing from 60 °C for neat PHBV up to 75 °C for the 45 wt% composite) and a longer cooling time (30 s). These processing conditions provided sufficient thermal energy and time for the polymer chains to organize into a semi-crystalline structure before ejection. Consequently, the injection-molded parts possess a semi-crystalline structure, which explains their high stiffness (Young’s modulus of approximately 4.5 GPa) and the absence of rubbery behavior at room temperature, despite the glass transition temperature (*T*_mg_) being near 0 °C.

Additionally, the second heating scan of neat PHBV and the biocomposites containing 15 and 30 wt% SSH shows, in addition to cold crystallization, the presence of endothermic melting peaks. Neat PHBV exhibits a bimodal melting pattern, which has been reported previously in ref. [78]. This behavior is associated with the melting and reorganizing of crystals into new crystals with higher structural perfection, which are more stable and melt at higher temperatures. Both crystal forms share the same structure; however, the first, lower-temperature-melting form, is characterized by a smaller lamellar thickness than the more stable crystals melting at higher temperatures [78].

Regarding *X*_c_ variations, PHBV–SSH15 biocomposites did not indicate significant differences compared to neat PHBV. These findings contrast with results reported in other works for aliphatic polyesters in which cellulosic fillers typically act as nucleating agents and cause notable changes in thermal and crystallization behavior [79,80]. In the present case, such an effect is not evident, leading to the conclusion that the addition of 15 wt% cellulose fibers has only a minimal impact on the melting and crystallization behavior of this PHBV grade. In contrast, the biocomposites containing 30 and 45 wt% show the absence of crystalline structure. For the PHB–SSH30 sample, the endothermic peak observed during the second heating corresponds to the melting of crystals formed only during the cold crystallization in the second heating cycle. The DSC curve of the second heating scan for the PHBV–SSH45 sample exhibits no endothermic melting peak.

The variation in characteristic glass transition temperatures, i.e., extrapolated onset temperature (*T*_eig_), midpoint temperature (*T*_mg_), and extrapolated end temperature (*T*_efg_), as well as the glass transition temperature step height (Δ*c*_p_) of PHBV and biocomposites derived from the second heating scan, are also presented in Table 6. The glass transition was detected only for neat PHBV and PHBV–SSH15, showing a decrease in *T*_mg_ from −0.2 to −12.0 °C. Although lignocellulosic fillers typically act as nucleating agents—as observed for the 15 wt% loading—a different mechanism dominates at higher filler contents (30–45 wt%). The complete suppression of crystallization during cooling can be attributed to the confinement effect and severe restriction of molecular mobility. The excessive volume fraction of rigid SSH particles creates a physical barrier that hinders the folding of PHBV chains into crystalline lamellae. Furthermore, strong interfacial interactions were detected via FTIR between PHBV carbonyls and SSH hydroxyls. These likely act as anchoring points, chemically immobilizing the polymer chains. Consequently, despite the potential nucleation sites provided by the filler, the high viscosity and restricted chain dynamics prevent crystal growth within the experimental cooling timeframe of 10 °C/min.

As a result, at the applied cooling rate, the polymer chains are unable to organize into a crystalline structure. Overall, these findings indicate that adding 15 wt% SSH does not cause substantial changes in thermal behavior compared to neat PHBV, whereas increasing the filler content above 15 wt% leads to a strong suppression of crystallinity from the melt under the applied DSC cooling conditions.

### 3.7. Uniaxial Tensile Tests

**Figure 9:**
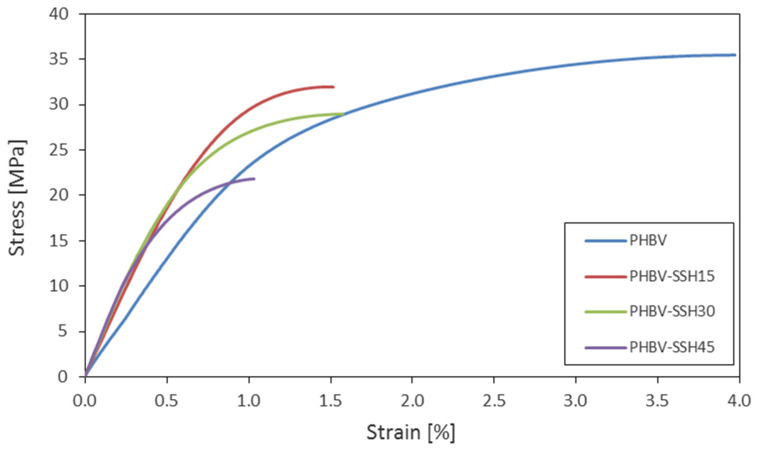
Stress–strain curves for all tested materials were obtained in the uniaxial tensile test.

The representative stress–strain curves for both pure PHBV and PHBV–SSH composites obtained in the uniaxial tensile test are presented in Figure 9. It can be observed that none of the composites exhibit a well-defined yield point. The stress–strain curves and the results presented in Table 7 reveal significant differences in mechanical behavior between the pure PHBV and the PHBV–SSH biocomposites. For pure PHBV, the curve displays characteristics typical of semi-crystalline thermoplastics. Following the initial linear-elastic region, a smooth transition into the plastic deformation zone is observed, ending with fracture at an elongation of approximately 4% and a maximum tensile stress of about 35 MPa. This suggests a quasi-brittle fracture mechanism, often observed in unmodified aliphatic polyhydroxyalkanoates, where deformation is limited by the premature failure of the matrix before significant structural reorganization can occur [81].

The addition of lignocellulosic particles results in significant changes in the deformation behavior. The tensile curves exhibit a more undulating slope in the elastic region, and the plastic zone shortens. An increase in the tensile modulus from approximately 2.6 GPa to 4.1 GPa was noted for 15 wt% content. Although a slight decrease in tensile strength from approximately 35 MPa to 32 MPa was observed, the lignocellulosic phase still acts as an effective microreinforcement, restricting mobility of polymer chain segments and enabling stress transfer at phase boundaries; the overall reduction in strength is likely due to imperfect interfacial adhesion or local stress concentrations. The increase in stiffness is the effect of both the mechanical stiffening of the matrix and the better dissipation of strain energy in the presence of particles with a high tensile modulus.

The increase in the SSH content to 30% results in a growth of the tensile modulus to approximately 4.4 GPa while simultaneously decreasing the tensile strength to 29 MPa and the elongation at break to approximately 1.5%. The contribution of the rigid phase reaches a critical level in this range, and the phase boundaries become the dominant sites of the crack initiation, limiting the ability to transfer plastic deformations. Nevertheless, the structure remains relatively homogeneous, as evidenced by the low values of the standard deviation (approximately 3% for the modulus and 0.5% for the tensile strength).

The tensile modulus further increased to approximately 4.5 GPa for the highest filler content (45%), indicating the dominant effect of stiffening. It is also accompanied by a substantial decrease in tensile strength (to approximately 21 MPa) and elongation at break to a value of approximately 1%. The stress–strain curves indicate the absence of a yield phase and a quasi-brittle material response, which can be associated with the excessive densification of the lignocellulosic phase and the formation of microdefects at the phase boundaries. The phenomenon is characteristic of biocomposites containing high loadings of plant-based fillers. In this regime, the interactions between particles become the dominant mechanism, and the adhesion to the matrix is reduced.

The variation in the tensile modulus and tensile strength values within the examined series is relatively small, confirming good homogeneity of the samples and high repeatability of the injection-molding process. The coefficient of variation (v) for the tensile modulus does not exceed 4.3% for pure PHBV and 3% for PHBV–SSH biocomposites. These values are in the range of 1–2% for tensile strength. The increase in variation to 5–8% is observed for the biocomposites containing 30–45 wt% SSH. This is a natural behavior for particulate composites; it results from local particle agglomeration, non-uniform wetting of fiber surfaces, and microdefects formed during the injection-molding process.

The results obtained in the research showed that the stiffness of the composites increases with the growth of SSH content. The tensile strength and elongation at break decreased with an increase in SSH loadings. It confirms the relationship between structural reinforcement and the loss of ductility, which is a typical phenomenon for PHBV biocomposites containing lignocellulosic plant particles. The results are consistent with observations by other researchers who examined PHBV composites containing lignocellulosic fillers. The results obtained in this study are fully consistent with the observations reported by Sánchez-Safont et al. [82]. They analyzed the influence of the content of natural cellulosic fillers derived from sawdust and nutshells on the mechanical and thermal properties of PHBV composites. The results have shown that the application of lignocellulosic particles to the composites caused a significant increase in the Young’s modulus and the overall stiffness of the material. It is caused by restricted polymeric chain mobility and the reinforcing effect of the material. A simultaneous decrease in tensile strength and elongation at break was also reported. Although FTIR analysis suggests an interaction between SSH and PHBV and possible formation of hydrogen bonds between the hydroxyl groups of SSH and the carbonyl groups of PHBV, the decrease in tensile strength might suggest that these chemical interactions are insufficient to counterbalance the structural effects for higher filler loadings. This confirms that a geometric effect—specifically, the role of rigid particles as stress concentrators—dominates over chemical interface strengthening. Consequently, at higher filler contents (30–45 wt%), matrix discontinuity and potential particle agglomeration limit effective stress transfer, outweighing the benefits of molecular-level bonding observed spectroscopically. Sánchez-Safont et al. [82] also reported that the excessive amount of the filler (above 30 wt%) led to degradation of the mechanical integrity and the increased brittle fracture behavior. They also highlighted the importance of the balance between stiffness and interfacial adhesion to maintain the favorable mechanical properties of biocomposites. A similar effect was noted in this work. Increasing SSH content resulted in a gradual growth of tensile modulus and stiffness, simultaneous with a decrease in elongation at break and a noticeable transition of the material to a more brittle character. The results obtained in this research confirm the relationship between the amount of the lignocellulosic filler and the mechanical properties of the biopolymers. They indicate the existence of an optimal filler content (~15–30%) at which a balance between stiffness and ductility of the material is achieved.

Similar changes in the material’s mechanical properties were noted by Fortunati et al. [83] who examined PLA composites modified using cellulose nanocrystals (CNC) and plasticized with limonene. They noted an increase in stiffness and a decrease in elongation at break after CNC incorporation. At the same time, the use of limonene as the natural plasticizer resulted in a decrease in the tensile modulus and a significant increase in ductility. It is probably associated with the reduction in glass transition temperature and the increased mobility of amorphous segments of the PLA chain. The synergistic effect was observed for ternary systems of PLA/limonene/CNC, in which the simultaneous presence of plasticizer and nanocellulose allowed for the retention of high flexibility with a moderate increase in the modulus. It is probably related to the nucleation effect and the reorganization of the amorphous-crystalline phase. The phenomena are consistent with the results obtained in this work. The changes in E, σb, and εb parameters indicate the relationship between the stiffening and plasticization effects, as well as the role of the filler and its interactions with the polymer phase.

The results presented here are also consistent with the observations of Reis et al. [84]. They analyzed the properties of PHB composites containing coffee husk (CH) and parchment particles (CP) as fillers. They reported that a low filler content (10%) did not significantly influence the tensile strength. An increase in the tensile modulus and a decrease in the specific energy were noted for the filler content of 20 wt%. The decrease in tensile strength suggests that the efficiency of load transfer between the matrix and the filler is limited at higher loadings. This behavior is typically attributed to the reduced interfacial area available for stress transfer and the potential formation of stress concentration points at the phase boundaries. The increase in stiffness results from limitations in the polymer chains’ mobility and the mechanical blocking of deformation by the lignocellulosic particles. Similar observations are noted in this work. The increase in SSH content in the PHBV matrix led to the growth of the tensile modulus (to 4.5 GPa), accompanied by a significant decrease in the elongation at break (to approximately 1%) and a reduction in the tensile strength. Similarly to the PHB–CH composites, PHBV–SSH samples exhibit a transition from the viscoplastic to the quasi-brittle behavior controlled by the propagation of microcracks at phase boundaries. The increase in lignocellulose particle content above 20% leads to deterioration of the interfacial integrity and reduction in the load transfer efficiency. It constitutes the primary mechanism responsible for the reduction in strength in PHBV–SSH biocomposites.

The increase in tensile modulus for moderate filler content (to 30 wt%) is caused by the rigid reinforcement effect associated with the addition of SSH particles, which act as micro-reinforcements within the polymer matrix. The fibrous–platelet morphology of the filler, which is characteristic of lignocellulosic cell-wall structures, promotes axial stress transfer. It results in mechanical stiffening of the composite. This mechanism is associated with the limited mobility of the polymer chain segments and the formation of local stress zones in which the lignocellulosic particles act as centers of the elastic deformation initiation [85,86].

As reported in ref. [86], the reinforcement efficiency of biofibers depends on their cellulose content and microfibril angle. The fibers with a low microfibril angle (<15°) exhibit high stiffness and high load-transfer efficiency. The sunflower seed hulls contain a significant amount of lignin and hemicellulose. The microstructure of the cell wall allows for partial flexibility. The balance between the increase in modulus and preservation of interfacial integrity is observed for the filler content of approximately 15–30%. Similar reinforcement effect has been observed in PLA and PHB composites containing hemp or rice fibers, in which the increase in tensile modulus of 20–40% is associated with the formation of local zones with the ordered particle orientation [86]. However, the overloading effect of the filler is observed for its higher loadings (above 45%). Paukszta and Borysiak [85] noted that the excessive lignocellulose content weakens the interfacial adhesion. The hydrophilic nature of the filler impedes its wetting by the nonpolar polymer matrix. Therefore, the decrease in the effective stress transfer is observed. The deformation is controlled by the initiation of microcracks and their propagation along the phase boundaries.

The increase in fiber content in the composite leads to its increased porosity and limits the matrix’s ability to compensate for the stresses through plastic deformation [86]. Paukszta and Borysiak [85] emphasized that the polymorphic morphology of the cellulose, especially the presence of its second type formed during the partial alkalization, reduces nucleation activity and might lead to a decrease in the mechanical strength of composites. In systems containing cellulose fibers classified to the first type, the load transfer is more efficient due to the greater parallelism of the microfibrils and the higher structural stiffness. As a result, the characteristic relationship between mechanical properties and content of the filler is observed for the PHBV–SSH composites. An initial increase in stiffness, which is a result of the structural reinforcement and fiber orientation, is observed. After that, the decrease in both tensile strength and impact tensile strength is noted for filler loadings of 30–40%. It is caused by the fact that adhesion and microspores begin to dominate over the reinforcement effect. The transition from viscoplastic to quasi-brittle fracture behavior results from the combined influence of morphological factors (microfibril orientation, lignin content, and porosity) and rheological factors (limited matrix flow around the particles).

The observed reduction in tensile strength at higher filler loadings (30–45 wt%) is most likely associated with the agglomeration of SSH particles and the saturation of the matrix capacity to wet the filler surface fully. These mechanisms can be indirectly inferred from the macroscopic mechanical response and the increased scatter of mechanical properties and are commonly reported for high-filled lignocellulosic biocomposites. Although microscopic analysis of the fracture surfaces (SEM) was not performed in this study, similar mechanisms have been well-documented for comparable high-filled lignocellulosic biocomposites [85,87,88]. While macroscopic trends clearly indicate a transition to quasi-brittle behavior, confirming the specific micromechanical failure modes (e.g., interfacial debonding versus fiber pull-out) would require further microscopic inspection.

### 3.8. Impact Tensile Strength

**Figure 10:**
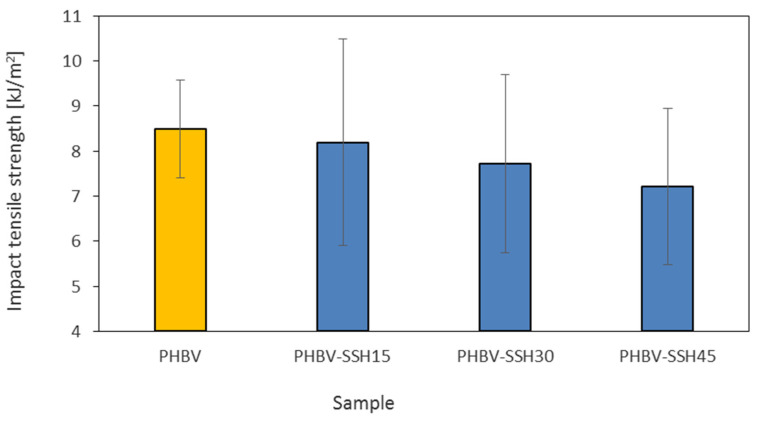
Impact tensile strength of all analyzed samples of biocomposites.

The resistance to cracking under dynamic loading conditions is a crucial functional parameter for biocomposites used for different technical applications, especially to produce thin-walled goods or components exposed to mechanical impacts. The impact tensile strength of biocomposites is presented in Figure 10. It depends on the material microstructure and the kind of interaction between the matrix phase and the filler particles, particularly for PHBV-based biopolymers, which exhibit a semicrystalline structure and limited ductility. Avérous and Digabel [87] reported that the lignocellulosic particles might act both as reinforcement agents and as crack initiators, depending on their wettability and adhesion to the polymer matrix.

The average fracture energy during impact tensile testing was approximately 8.5 kJ/m^2^ for pure PHBV. Application of SSH as the filler did not improve these results. The impact tensile strength values were approximately 8.2 kJ/m^2^ for PHBV–SSH15, 7.7 kJ/m^2^ for PHBV–SSH30, and 7.72 kJ/m^2^ for PHBV–SSH45. The differences between the values are relatively small. It can be seen that the material’s ability to absorb energy under the impact loading decreases with the increasing filler content in biocomposites. The decrease in the impact tensile strength values is associated with a change in the material behavior from viscoplastic to quasi-brittle, in which the phase boundaries are dominant. Rigid lignocellulosic particles contribute to reinforcing the stiffness of the material. However, under dynamic loading, the reduction in impact strength indicates that the rigid SSH particles are more likely to act as stress concentrators rather than energy absorbers. It can be hypothesized that at high filler contents (30–45 wt%), the continuity of the polymer matrix is disrupted, which facilitates crack propagation. Similar embrittlement mechanisms driven by limited interfacial adhesion have been noted for other PHBV-based composites [25,82,88].

For the PHBV–SSH15 sample, the tensile impact strength value is very similar to that of pure PHBV, despite the significant increase in tensile modulus. It can be related to the relatively good dispersion and sufficient adhesion at the phase boundary. The particles act mainly as centers for elastic energy dissipation. Limited slipping might contribute to energy absorption. The mechanisms are displaced by the premature crack initiation when the content of SSH exceeds approximately 30 wt%. The fracture energy and elongation at break decrease as the modulus simultaneously increases. The variability of results (deviations of 1.7–2.3 kJ/m^2^) is higher for biocomposites than for pure PHBV, which is characteristic of particle-filled materials. It is caused by the local variations in particle dispersion and orientation, as well as the presence of small gas or shrinkage voids formed due to the increased melt viscosity.

The results contained in this work are consistent with findings obtained by other researchers. Reis et al. [84] noted the lack of improvement or even the decrease in the impact tensile strength for composites containing 10–20 wt% coffee waste. It was a result of the poor adhesion and the formation of microvoids around the particles. Sánchez-Safont et al. [82] showed that the tensile modulus increases while the resistance-related properties, e.g., elongation and the impact strength, decrease with the increasing filler content for PHBV composites containing lignocellulosic waste (sawdust, shells). Frącz et al. [25] reported similar trends for the injection-molded PHBV composites with plant fibers. The increase in viscosity and porosity contributes to the lower impact strength, especially for high filler content. The generalized description of the mechanisms, including the role of filler hydrophilicity, microfibrillar angle, cellulose polymorphism, and quality of interfacial adhesion, was described by Paukszta and Borysiak [85]. They indicated the decrease in fracture energy under conditions of poor adhesion and the high lignocellulosic phase content. The results presented in this work are also consistent with the observations noted by Hejna et al. [88]. They emphasized that the dominant factor reducing the impact strength of lignocellulosic biocomposites is the discontinuity of the phase boundary structure. Boey et al. [89] indicated that the biological or chemical surface modifications of the fillers can improve the adhesion mechanism. Hasan et al. [19] highlighted that the dynamic crack propagation is related to the moisture content, porosity, and fiber orientation. These factors can modify the material’s resistance to brittle fracture.

### 3.9. Hardness

**Figure 11:**
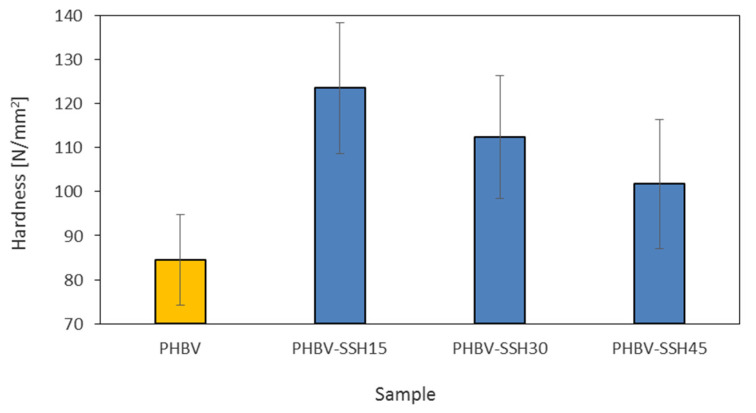
Hardness of specimens in the A region of samples.

**Figure 12:**
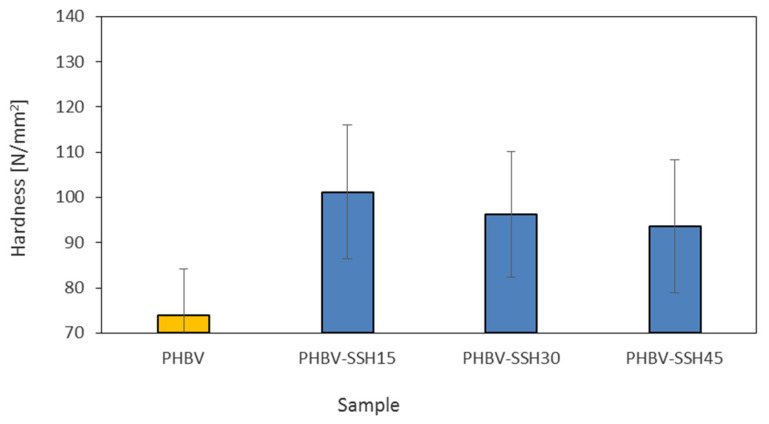
Hardness of specimens in the B region of the samples.

The hardness was measured in two specific injection areas of the samples: in the central part of the specimens (Region A) and in the end zones (Region B). This approach was adopted to evaluate material homogeneity along the flow path and to assess the influence of processing conditions (such as pressure drop and cooling rate variations) on the local mechanical properties. The results are presented in Figure 11 and Figure 12. For pure PHBV, the hardness was approximately 85 N/mm^2^ in the central area and 74 N/mm^2^ in the end zones, respectively. The addition of SSH caused a systematic increase in hardness reaching approximately 120 N/mm^2^ for PHBV–SSH15, 110 N/mm^2^ for PHBV–SSH30, and 100 N/mm^2^ for PHBV–SSH45 in the A region. Similar relationships were also observed in the B region with slightly lower absolute values.

These results show that the addition of SSH to the PHBV matrix effectively increased hardness. It is caused by the increased share of the rigid lignocellulosic phase in the material. A similar hardening effect was reported in a study concerning the production of PHBV–coffee composites [84]. The increase in hardness and tensile modulus was strongly associated with the crosslinking and improved dispersion of lignocellulosic particles. A growth of mechanical strength without a reduction in formability was also observed in studies related to the production of PHBV composites containing almond waste and rice husk at loadings of 10–20 wt% [90].

The highest hardness values were obtained for biocomposites containing 15 wt% SSH. A gradual decrease in hardness was observed for higher dosages of fillers (30 and 45 wt%). These phenomena are consistent with results obtained for PHBV composites containing lignocellulosic waste as a filler. A slight decrease in hardness observed for the highest filler content (45 wt%) implies a saturation effect, in which the continuity of the material is compromised. It can be related to difficulties in achieving full wetting of the particles by the matrix at such high-volume fractions. This phenomenon has also been reported for other highly filled biocomposites [82,91]. The increase in hardness for biocomposites with filler loadings of 10–20% was also noted for PLA and biochar, as well as for PLA and the coffee-ground biocomposites [92]. Similar results were also noted for TPU containing lignocellulosic fillers [93].

The middle part of the sample (region A) was characterized by a higher hardness than the end zones (region B) for all tested materials. This difference, typically 10–20 N/mm^2^ on average, is associated with the variations in thermomechanical history and cooling along the specimen. More stable conditions of crystallization and compression occur in the A region, which improves the formation of denser crystalline structure and better interfacial adhesion. Micropores and voids might occur in the B zone, which is caused by the lower fiber orientation and the local relaxation of pressure. Similar phenomena associated with the property gradient were observed in [94], in which the cooling and pressure parameters influence the hardness in biocomposites produced with the use of PP and rice husk.

The standard deviation of hardness is within the typical range for composites containing natural fillers (10–15 N/mm^2^). It is a result of microstructural inhomogeneity, e.g., fiber orientation, local porosity, and the skin effect. The differences in hardness between the pure PHBV and PHBV–SSH biocomposites are statistically significant (*p* < 0.05), which confirms surface hardening in agreement with previous studies on PHBV composites containing cellulose-based fillers [90,91]. From a practical point of view, the SSH content of approximately 15 wt% seems to be the most advantageous as it provides the highest hardness while maintaining a low risk of the formation of structural defects. The results obtained in this part of the work are the starting point for the further optimization of the injection molding parameters and for the production of PHBV-based biocomposites, which are characterized by sustainable mechanical and biodegradable properties.

### 3.10. Water Absorption Capacity

**Figure 13:**
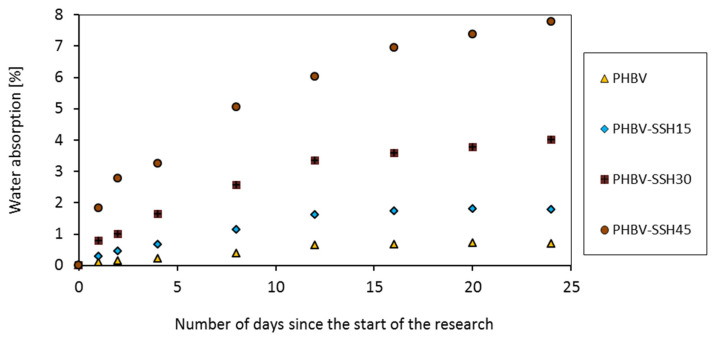
Water absorption capacity for all analyzed samples.

The water absorption measurement was carried out to determine the influence of SSH content on the hydrophilicity and moisture resistance of PHBV biocomposites. The results obtained in this work are presented in Figure 13. The water absorption value was very low for pure PHBV, reaching approximately 0.7% after 24 days. Two stages can be distinguished on the water absorption curve. A rapid increase in moisture was observed in the early stage, to the value of approximately 0.4% after 8 days. A pronounced plateau was noted later, which indicates that equilibrium has been reached. This behavior is characteristic of semi-crystalline aliphatic polymers, and it corresponds to the classical Fickian model in which diffusion occurs predominantly in the amorphous phase [95,96,97,98,99].

The addition of SSH filler resulted in a significant increase in both water absorption and equilibrium level. After 24 days, the water absorption reached approximately 1.8% for the PHBV–SSH15 sample, 4.0% for the PHBV–SH30 specimen, and 7.8% for the PHBV–SH45 sample. The biocomposite containing SSH exhibited a several-fold higher moisture increase compared to pure PHBV after just one day. It confirms the existence of additional capillary and interfacial transport routes. The complete plateau of the curves was not observed for samples with the highest filler content, which indicates that the equilibrium level was not reached. The shape of the sorption curves suggests a deviation from Fickian behavior, pointing to a more complex transport mechanism involving both polymer matrix and porous network/phase interfaces.

The increase in water absorption is a result of the synergistic interaction of several factors. Firstly, SSH contains significant amounts of cellulose, hemicellulose, and lignin, which have a lot of hydroxyl groups capable of forming hydrogen bonds with water. The significant increase in water uptake suggests the presence of additional transport pathways within the composite structure. This might be attributed to the hydrophilic nature of the filler itself and likely imperfect adhesion at the filler–matrix interface, which can create capillary channels that facilitate moisture diffusion. The presence of the filler can affect the matrix morphology by modifying its crystallinity. While increased crystalline ordering generally reduces water permeability, this effect is often counterbalanced by enhanced porosity and the formation of diffusion channels in composites with higher filler loadings. The molecular binding mechanisms and the uniform dispersion predominate in samples with well-dispersed particles (15 w% SSH). In samples containing 30–45% SSH, the filler agglomerates are formed, leading to the formation of continuous transport channels and the increased sorption rate.

Water absorption increases with increasing SSH content due to the hydrophilic nature of the filler. The water absorption of natural fillers contained in biopolymers can lead to the formation of voids and microcracks at the filler–matrix interface region, which might result in a reduction in the mechanical properties and dimensional stability of biocomposites.

The addition of natural fillers into the polymer matrix results in an increase in water absorption, which leads to interfacial degradation, which is the primary cause of structural and mechanical failure of biocomposites. This interfacial degradation process is caused by multiple mechanisms. The addition of natural fillers results in differential swelling between the rigid filler and the polymer matrix, which leads to the formation of voids and microcracks at the interface. Water molecules travel along these newly formed gaps and voids through capillary action, further accelerating moisture penetration. It leads to ultimate debonding and loss of stress transfer efficiency [99,100].

The water absorption of biopolymers and their influence on dimensional stability and interfacial degradation should be included during the design and production of goods. The application of biopolymers with natural fillers for the production of items for which dimensional stability is crucial might be limited and should be preceded by in-depth and comprehensive studies and their analysis. For example, in high-humidity environments or when exposed to water (e.g., outdoor applications, underwater components, etc.), this material may require proper protection (e.g., moisture protection coating). But in moderate conditions (e.g., normal air humidity, no continuous contact with water), an increase in water absorption to the level of several percent of the mass does not necessarily disqualify the material. For example, for a composite with 15% SSH, the absorption of approximately 1.8% (after 24 days of immersion) is an acceptable value in many applications, especially if very high dimensional tolerances are not required.

The water absorption process is a complex phenomenon in composites containing lignocellulosic filler. The molecular diffusion, capillary transport, and microstructural degradation mechanisms occur simultaneously. Choy et al. [98] reported that the water absorption occurs in accordance with Fick’s law for the phenolic composites containing lignocellulosic fillers, e.g., rice husk or coconut shell powder. It increases rapidly as a function of the square root of time and then reaches a stable equilibrium. The value of the kinetic exponent n nearly 0.5 indicates a classical Fickian diffusion mechanism. The water adsorption mechanism becomes more complex for the higher filler content of above 40–50 wt%. The water absorption leads to the swelling of lignocellulosic particles and the formation of microcracks in the matrix, which create the capillary pathways and facilitate further water transport. As a result, this mechanism becomes complex, i.e., Fickian in the polymer phase and non-Fickian in the network of pores and in the interfacial cracks [89]. Similar phenomena were noted by Le Duigou et al. [99] who examined the long-term immersion of PLA/flax composites in seawater. They reported that water absorption induces microporosity, fiber swelling, and localized adhesion loss, leading to an increase in water uptake of up to 200–300%, in comparison to pure PLA. Water infiltrates along the fibers with increasing exposure time, which promotes the propagation of microcracks and degradation of mechanical properties. This behavior is also characteristic of other biocomposites in which plant-derived fillers act as diffusion channels.

Deroine et al. [101] reported that water absorption in PHBV biocomposites follows a second-type Fickian diffusion model (Fick II). The diffusion rate depends on the fraction of the crystalline phase. The crystalline regions impede water penetration, whereas diffusion predominantly takes place within the amorphous phase. Water acts as a plasticizer, leading to a decrease in the glass transition temperature and the formation of localized hydrostatic stresses. It results in the long-term degradation of the matrix. Authors also observed that under prolonged water immersion, there is a gradual mass loss and a decrease in mechanical strength by 30–40%. Fortunati et al. [83] and Arrieta et al. [102] emphasized that the presence of cellulose fibers or nanoparticles in biodegradable matrices (PLA, PHB, PHBV) increases both overall hydrophilicity and volume of interfacial pores. For moderate filler contents below 20%, water binding is primarily governed by molecular mechanisms, with hydrogen bonding occurring between the hydroxyl groups of the filler and the carbonyl groups of the matrix. The capillary component resulting from the presence of microcapillaries and microscale interfacial gaps occurs for a higher filler content of above 30%. As a result, sorption appears to occur in two stages, where the initial diffusion phase likely transitions into a non-Fickian behavior associated with the relaxation and potential degradation of the composite microstructure [101,102].

Choy et al. [98] and Le Duigou et al. [99] also reported that water absorption not only increases water content but also contributes to the deterioration of adhesion and the development of micropores. As a result, an increase in diffusion coefficient and a reduction in time required to reach saturation are observed. The diffusion coefficient values for lignocellulosic composites can increase by an order of magnitude when the filler content increases from 30% to 55%, which underscores the predominant role of microstructural transport channels. In terms of material stability, Deroine et al. [101] indicated that prolonged exposure to water leads to the progressive hydrolytic degradation of PHBV, resulting in a decline in its mechanical properties. Additionally, Fortunati et al. [83] and Arrieta et al. [102] observed that the presence of cellulose accelerates the process by facilitating the moisture absorption and causing the local dissolution of the polymer chain network.

The results obtained in this work are consistent with the observations and confirm the specific behavior of lignocellulosic biocomposites, in which an increase in the content of the hydrophilic phase leads to a reduction in water resistance. It is worth highlighting that low SSH content (approximately 15% here) does not cause significant deterioration of material stability while simultaneously increasing stiffness and hardness. These filling proportions can be considered as a compromise from the perspective of balance between mechanical properties and moisture resistance. On this basis, PHBV–SSH15 can be considered the most balanced formulation for applications exposed to intermittent moisture.

### 3.11. Shrinkage

**Figure 14:**
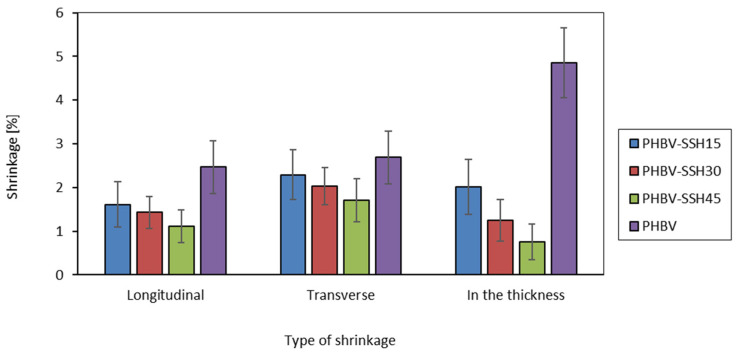
Shrinkage in thickness, in longitudinal and transverse directions.

The longitudinal and transverse shrinkage, as well as the shrinkage in the thickness, were analyzed for pure PHBV and for PHBV–SSH biocomposites (Figure 14). The longitudinal shrinkage was measured in line with the flow direction in the mold cavity. The transverse shrinkage was analyzed in the plane of the mold and perpendicular to the flow direction. The measurement system captures the strain anisotropy, which is a result of the macroparticles’ orientation, flow line patterns, and the temperature gradients during the solidification. The longitudinal shrinkage is a result of the elastic strain relaxation formed during the shear and elongation of the melt during cavity filling. However, in the transverse direction, the dominant effects are thermal shrinkage and crystallization shrinkage, which are less dependent on molecular chain orientation. The highest values of the shrinkage are noted in the direction of thickness because the strongest cooling gradient occurs between the cold mold walls and the warmer core of the molded part. In this direction, the material cannot flow freely, and part of the retraction accumulates as through-thickness shrinkage, which promotes sink marks and surface deformations.

The shrinkage values for pure PHBV confirm the classical anisotropy. The longitudinal shrinkage was approximately 2.5%, while the shrinkage in the thickness was nearly 5%. It indicates that the material shrinks more than twice as much in the normal direction as it does in the plane. It is a characteristic behavior for semi-crystalline polymers with a high degree of crystalline content and a relatively low thermal conductivity. The core of the sample remains in a molten state for a long time under conditions of slow heat removal. The delayed crystallization leads to secondary shrinkage, which occurs after ejection from the mold. Similar behavior has been observed in biocomposites based on PHBV and PLA containing different natural fibers. The high crystalline fraction and the significant volumetric transition cause large processing-induced deformations when the material is not properly stabilized with additives or fibers [103,104].

The addition of SSH influences the shrinkage values significantly. The longitudinal shrinkage decreases to approximately 1.5% for PHBV–SSH15, which represents a reduction of nearly one-third compared to pure PHBV. The transverse shrinkage is reduced from nearly 3% to slightly above 2%. The shrinkage in thickness decreases from 5% to slightly over 2%, corresponding to a reduction of nearly 50%. With the further increase in SSH content to 30%, the shrinkage continues to decrease in the thickness direction, reaching approximately 1.25%. For 45% SSH content, it decreases to below 1%. It represents a reduction in shrinkage by roughly 80% in comparison to pure PHBV. In the mold plane, the same trend is observed. Although the magnitude of changes is smaller. The longitudinal and transverse shrinkage remain at approximately 1–1.7% for 45% SSH content, which corresponds to a reduction of 30–40% in comparison to pure PHBV. The anisotropy between directions diminishes, showing that composites with high SSH content exhibit higher dimensional isotropy than the neat matrix.

The further increase in SSH content results in a continued decrease in the shrinkage, reaching approximately 12.25% in the thickness for 30 wt% and below 1% for 45 wt%. It corresponds to a shrinkage reduction of approximately 80% in comparison to pure PHBV. A similar relationship was observed in the plane of the mold. For the maximum tested filler content (45 wt%), longitudinal and transverse shrinkage remain at approximately 1–1.7%, corresponding to a 30–40% reduction in comparison to pure PHBV.

The lignocellulosic phase acts as internal reinforcement, which anchors the matrix and restricts its free contraction during cooling and crystallization [103,104]. This mechanism is described in many works concerning biocomposites produced using PHBV and PLA containing filler plants. Bledzki and Jaszkiewicz [103] showed that an increase in fiber content leads to a significant increase in tensile modulus and an improvement in dimensional stability of the components compared to reference systems based on PP. This effect is associated with the formation of a fibrous network within the material volume. Zini et al. [104], in their study on PHBHHx (poly(3-hydroxybutyrate-co-3-hydroxyhexanoate)) biopolymers containing plant fibers, pointed out that the fibrous phase limits both shrinkage during molding and deformation during service at elevated temperatures. This characteristic enables biocomposites to compete with conventional engineering materials [104].

For PHBV–SSH biocomposites, the reduction in shrinkage is associated with several specific overlapping mechanisms. Firstly, the particles and fiber fragments, which possess a relatively high tensile modulus, absorb a portion of the thermal and crystallization stresses generated in the matrix during cooling. These fibers are less prone to retraction than the polymer phase, which helps reduce the overall volumetric shrinkage. Secondly, the presence of the solid phase significantly disrupts the ability of the PHBV chains to undergo further reorganization after passing through the crystallization temperature. Some segments of the polymer chains, trapped between the filler particles, are unable to fully integrate into the ideal crystalline lattice. As a result, these segments do not contribute to crystallization-induced shrinkage, leading to a reduction in overall volumetric shrinkage during cooling and crystallization. This phenomenon has been well described for various PLA-based biocomposites containing plant-derived fillers, in which the fibers act as nucleating agents while simultaneously limiting the growth of large spherulites and reducing secondary retraction [105,106].

The reduction in the shrinkage depends on direction. In the thickness direction, the SSH particles act as bridges connecting the layers adjacent to the mold walls with the core of the sample. The fibers limit the material’s tendency to sink towards the core by functioning as a reinforcing phase that counteracts the internal shrinkage induced by the temperature gradient during cooling. In the plane of the mold, part of the chain orientation along the flow direction becomes “anchored” by the irregularly distributed particles, which reduces the relaxation of elastic deformations. However, this effect is weaker than in the thickness direction because the flow and the influx of the material during compression can still compensate for some of the linear retraction. It explains why a relative reduction in the shrinkage is the greatest in the thickness direction and the smallest in the transverse direction.

The essential complement to the quantitative analysis is the assessment of the dissipation of the results obtained. The standard deviation is relatively high for pure PHBV, especially in the thickness direction. It indicates a significant sensitivity of the material to local variations in cooling conditions and potential changes in mold temperature. The standard deviation decreases for biocomposites containing SSH as a filler, especially with its high content. It shows that the lignocellulosic phase not only reduces the average shrinkage but also stabilizes the material response, making it less sensitive to minor process variations. Similar results are reported in other works [103,107,108] for biocomposites containing various plant waste, in which the presence of plant fibers and particles improves dimensional stability and reduces susceptibility to warping.

From the practical point of view, the results obtained show that the addition of SSH to PHBV can significantly simplify the design of injection molding cavities. For pure PHBV, predicting a shrinkage of several percent in the thickness direction would be necessary, requiring appropriately oversized mold features and potentially leading to batch-to-batch variability. At the same time, the acceptable anisotropy between directions is maintained, reducing the risk of warping or distortion of the molded parts. However, it is important to note that such a high filler content also alters other material properties, including the tensile modulus, impact resistance, and water absorption. It requires a holistic approach to both composite design and processing conditions.

## 4. Summary and Conclusions

This study investigated the potential use of sunflower seed hulls (SSH) as a sustainable lignocellulosic filler in PHBV-based biocomposites. The research confirmed that SSH can be effectively incorporated into the polymer matrix at loadings of 15–45 wt%, obtaining homogeneous materials suitable for processing via extrusion and injection molding. The addition of ground SSH significantly altered the rheological behavior of the melt, requiring specific adjustments in the temperature profile and holding pressure in order to ensure stable processing. While the biocomposites produced exhibited a uniform structure, the higher filler loadings (30–45 wt%) resulted in increased viscosity and a transition towards a more complex, non-linear flow behavior.

Mechanical testing demonstrated a clear trade-off between stiffness and ductility characteristics of fiber-filled biocomposites. The tensile modulus increased significantly from 2.6 GPa for pure PHBV to approximately 4.5 GPa for the biocomposite containing 45 wt% SSH. It confirms the reinforcing effect of the rigid lignocellulosic particles. However, this improvement was accompanied by a reduction in tensile strength and elongation at break. As a result, the material exhibits a brittle nature and therefore, might be dedicated for the production of lightly loaded products. However, a full explanation of the phenomena requires further microscopic analysis, e.g., SEM analysis.

The surface hardness was also improved, reaching its maximum value for 15 wt% SSH loading. The notable deviation from typical nucleation behavior was observed in the thermal properties of biocomposites. Contrary to the effect observed at lower loadings, higher SSH contents (30–45 wt%) resulted in the complete suppression of crystallization during cooling under the applied DSC conditions. This phenomenon is attributed to the confinement effect and the restricted mobility of the polymer chains resulting from the high filler volume fraction and the interfacial interactions (consistent with hydrogen bonding) between PHBV and SSH identified during the FTIR analysis. Nevertheless, the molded parts exhibit a semicrystalline structure due to the injection-molding conditions and subsequent aging, despite the absence of crystallization during rapid cooling in the DSC analysis. This discrepancy arises because the dynamic cooling conditions in DSC analysis differ significantly from the conditions present during injection molding. Therefore, the crystallization in DSC analysis under laboratory conditions (cooling at 10 °C/min) might not be registered. Thermal stability analysis (TGA) also confirmed that despite a slight reduction in decomposition onset temperature, the biocomposites maintain a wide processing window with a safety margin of over 80 °C above the processing temperature, ensuring suitability for standard injection molding operations.

The key practical advantage identified in this study is the exceptional reduction in processing shrinkage. The addition of SSH reduced the shrinkage in the thickness direction by approximately 80% compared to pure PHBV. Conversely, the presence of hydrophilic lignocellulosic particles led to an increase in water absorption. The kinetics of this process suggest a complex mechanism involving both diffusion into the matrix and transport through the capillary network at the filler-matrix interface, particularly at higher filler loadings. The high-dimensional stability and water absorption are key advantages for the precise production of different components, e.g., flat protective covers, housings, and unreinforced castings.

All in all, the PHBV–SSH biocomposites, particularly with those with a filler content of 15–30 wt%, provide an optimal balance of high stiffness, improved hardness, and excellent dimensional stability. These properties, in conjunction with the environmentally friendly nature of the constituents, make this material a promising alternative for engineering applications in sectors requiring sustainable solutions with high technical performance.

## Figures and Tables

**Table 1:** Parameters of the extrusion process.

Temperature [°C]				Rotational Speed of the Extruder Screw [rpm]
Head	Zone 3	Zone 2	Zone 1	
PHBV				
160	160	155	145	100
PHBV-SSH15				
165	160	155	150	30
PHBV-SSH30				
170	165	160	155	40
PHBV-SSH45				
175	170	165	160	50

**Table 2:** Technological parameters of injection molding of specimens dedicated to the uniaxial loading test.

Parameter	Biocomposites			
	PHBV	PHBV–SSH15	PHBV–SSH30	PHBV–SSH45
Mold temperature [°C]	60	65	70	75
Melt temperature [°C]	167	170	175	180
Cooling time [s]	25	30	30	30
Packing time [s]	25	30	30	30
Packing pressure [MPa]	30	45	45	45
Injection speed [cm^3^/s]	35	35	35	35

**Table 3:** The characteristics of TG and DTG curves for the degradation steps of PHBV, SSH, and PHBV/SSH biocomposites.

Samples	*T*°_1_/*T*°_2_ [°C]	*T*_max1_/*T*_max2_ [°C]	*R*_max1_/*R*_max2_ [% min^−1^]	Δ*m*_1_/Δ*m*_2_ [%]	*m*_f_ [%]
PHBV	279	294	48.7	0	0
PHBV–SSH 15	268/320	278/338	63.7/0.8	91.4/5.7	2.9
PHBV–SSH 30	264/319	272/341	65.7/2.2	79.4/13.2	7.3
PHBV–SSH 45	261/317	267/343	52.2/2.9	67.9/21.1	10.9

**Table 4:** The characteristics of TG and DTG curves for the degradation steps of SSH.

	*T*°_1_/*T*°_2_ [°C]	*T*_max1_/*T*_max2_ [°C]	*R*_max1_/*R*_max2_ [% min^−1^]	Δ*m*_1_/Δ*m*_2_ [%]	*m*_f_ [%]
SSH	181/297	206/342	0.9/7.5	3.9/64.4	26.7

**Table 5:** DSC data of PHBV–SSH biocomposites obtained from the first heating scan and cooling scans.

Sample	First Heating Scan					Cooling Scan			
	*T*_m_ [°C]			−Δ*H*_m_ [Jg^−1^]	*X*_c_ [%]	*T*_c_ [°C]			Δ*H*_c_ [Jg^−1^]
	*T*_eim_	*T*_pm_	*T*_efm_			*T*_eic_	*T*_pc_	*T*_efc_	
PHBV	169.3	175.2	180.7	81.31	55	76.2	59.2	39.3	50.44
PHBV–SSH15	163.3	177.0	184.6	71.12	57	78.2	63.8	33.6	43.34
PHBV–SSH30	166.9	175.7	181.4	60.90	59	-	-	-	-
PHBV–SSH45	163.1	173.1	178.9	47.03	58	-	-	-	-

**Table 6:** DSC data of PHBV–SSH biocomposites obtained from the second heating scan.

Sample	Second Heating Scan												
	*T*_g_ [°C]			Δ*c*_p_ [Jg^−1^ °C ^−1^]	*T*_c_ [°C]			Δ*H*_cc_ [Jg^−1^]	*T*_m_ [°C]			−Δ*H*_m_ [Jg^−1^]	*X*_c_ [%]
	*T*_eig_	*T*_mg_	*T*_efg_		*T*_eicc_	*T*_pcc_	*T*_efcc_		*T*_eim_	*T*_pm_	*T*_efm_		
PHBV	−1.6	−0.2	2.1	0.18	42.9	49.7	54.9	10.41	155.8	162.1	167.4	83.21	50
PHBV–SSH15	−14.1	−12.0	−9.5	0.07	29.7	42.5	54.4	4.262	119.7	146.3	157.2	66.62	50
PHBV–SSH30	-	-	-	-	35.6	47.5	61.0	22.83	88.5	118.6	126.1	23.38	0
PHBV–SSH45	-	-	-	-	-	-	-	-	-	-	-	-	-

**Table 7:** Results of the uniaxial tensile test.

Material	Parameter	E_t_ [MPa]	σ_M_ [MPa]	ε_M_ [%]
PHBV	X*	2617.37	35.48	4.12
	S**	112.02	0.86	0.15
	V***	4.28	2.42	3.63
PHBV-SSH15	X	4061.9	31.89	1.49
	S	104.67	0.35	0.05
	V	2.58	1.10	3.38
PHBV-SSH30	X	4437.85	28.88	1.47
	S	125.82	0.14	0.08
	V	2.84	0.50	5.27
PHBV-SSH45	X	4545.55	21.12	1.07
	S	117.98	0.45	0.09
	V	2.60	2.14	8.49

X*—mean, S**—standard deviation, V***—variation coefficient.

## Data Availability

The original contributions presented in this study are included in the article. Further inquiries can be directed to the corresponding authors.
